# Structural Characterization of Two Short Unspecific Peroxygenases: Two Different Dimeric Arrangements

**DOI:** 10.3390/antiox11050891

**Published:** 2022-04-30

**Authors:** Dolores Linde, Elena Santillana, Elena Fernández-Fueyo, Alejandro González-Benjumea, Juan Carro, Ana Gutiérrez, Angel T. Martínez, Antonio Romero

**Affiliations:** 1 Centro de Investigaciones Biológicas “Margarita Salas” (CIB), CSIC, E-28040 Madrid, Spain; lolalinde@cib.csic.es (D.L.); esh@cib.csic.es (E.S.); jcarro@cib.csic.es (J.C.); 2 Intravacc, 3721 MA De Bilt, The Netherlands; elena.fueyo@intravacc.nl; 3 Instituto de Recursos Naturales y Agrobiología de Sevilla (IRNAS), CSIC, E-41012 Seville, Spain; a.g.benjumea@irnas.csic.es (A.G.-B.); anagu@irnase.csic.es (A.G.)

**Keywords:** unspecific peroxygenases (UPO), *Collariella virescens*, *Marasmius rotula*, crystal structures, analytical ultracentrifugation, dimeric arrangements, fatty acid epoxidation

## Abstract

Unspecific peroxygenases (UPOs) are extracellular fungal enzymes of biotechnological interest as self-sufficient (and more stable) counterparts of cytochrome P450 monooxygenases, the latter being present in most living cells. Expression hosts and structural information are crucial for exploiting UPO diversity (over eight thousand UPO-type genes were identified in sequenced genomes) in target reactions of industrial interest. However, while many thousands of entries in the Protein Data Bank include molecular coordinates of P450 enzymes, only 19 entries correspond to UPO enzymes, and UPO structures from only two species (*Agrocybe aegerita* and *Hypoxylon* sp.) have been published to date. In the present study, two UPOs from the basidiomycete *Marasmius rotula* (r*Mro*UPO) and the ascomycete *Collariella virescens* (r*Cvi*UPO) were crystallized after sequence optimization and *Escherichia coli* expression as active soluble enzymes. Crystals of r*Mro*UPO and r*Cvi*UPO were obtained at sufficiently high resolution (1.45 and 1.95 Å, respectively) and the corresponding structures were solved by molecular replacement. The crystal structures of the two enzymes (and two mutated variants) showed dimeric proteins. Complementary biophysical and molecular biology studies unveiled the diverse structural bases of the dimeric nature of the two enzymes. Intermolecular disulfide bridge and parallel association between two α-helices, among other interactions, were identified at the dimer interfaces. Interestingly, one of the r*Cvi*UPO variants incorporated the ability to produce fatty acid diepoxides—reactive compounds with valuable cross-linking capabilities—due to removal of the enzyme C-terminal tail located near the entrance of the heme access channel. In conclusion, different dimeric arrangements could be described in (short) UPO crystal structures.

## 1. Introduction

Unspecific peroxygenases (UPOs) are enzymes catalyzing the oxyfunctionalization of both aromatic and aliphatic molecules [1,2,3], including specific products that are difficult to be obtained only by chemical means. Therefore, they are of high biotechnological interest [4,5,6,7] for the regio- and/or stereoselective synthesis of biobased compounds and other fine chemicals and pharmaceuticals. These enzymes form a group of fungal heme-thiolate peroxidases (HTPs). Hence, they share a proximal cysteine residue acting as the fifth ligand of the heme iron with the phylogenetically distant cytochrome P450 monooxygenases (P450s) [8], present in all living organisms. This results in similar reaction chemistry but UPOs, as self-sufficient secreted peroxygenases, catalyze selective oxygenation reactions with advantages over P450s [9]. The latter often require an auxiliary flavin-containing enzyme/module and a source of reducing power, and are less stable proteins due to their intracellular nature.

Structural–functional information is required for the rational engineering of UPOs to optimally catalyze selective hydroxylations and epoxidations of interest. Since the emergence of the first P450 crystal structures [10], many thousands of P450 entries have accumulated during the 50 years of history [11] of the Protein Data Bank (PDB, www.rcsb.org, accessed on 1 March 2022). In contrast, although Hofrichter and coworkers [12] reported (from BLAST search in November 2021) the presence of over 4000 putative UPO/HTP encoding genes in the NCBI database and over 8000 in the sequenced fungal genomes available at Mycocosm (JGI, DOE), only 19 PDB entries refer to UPO enzymes (including complexes with different ligands) and UPO structures from only two fungal species have been published to date. These published structures correspond to: (i) the wild (i.e., nonrecombinant) [13,14] and yeast-evolved (and in vitro deglycosylated) [15] UPO from the basidiomycete *Agrocybe aegerita* (a synonym of *Cyclocybe aegerita*; *Aae*UPO), the first UPO described eighteen years ago [16]; and (ii) the very recently reported UPO of the ascomycete *Hypoxylon* sp (r*Hsp*UPO) obtained by yeast expression (and subsequent deglycosylation) of a putative UPO gene [17] (corresponding to protein ID 467810 of the *Hypoxylon* sp EC38 genome v3.0 sequenced at JGI, DOE). Moreover, for many years, the related chloroperoxidase of the ascomycete *Leptoxyphium fumago* (syn. *Caldariomyces fumago*) was the only HTP enzyme whose structure was known [18,19].

This scarcity of structural information is largely due to: (i) the limited number of wild UPOs available from fungal cultures, which basically includes those of the basidiomycetes *Coprinellus radians* [20], *Coprinopsis verticillata* [21], *Marasmius rotula* (*Mro*UPO) [22], *Marasmius wettsteinii* (*Mwe*UPO) [23] and *Candolleomyces aberdarensis* [24], and the ascomycete *Chaetomium globosum* [25], together with the above *Aae*UPO and the *L. fumago* chloroperoxidase—and (ii) the lack of adequate hosts for converting putative UPO sequences into active proteins [12]. The latter was first addressed by directed evolution of *Aae*UPO to increase the very low secretion levels by *Saccharomyces cerevisiae* [26], and permitted significantly higher production by transferring the evolved gene (with five mutations in the mature protein sequence) into *Pichia pastoris* [27]. Interestingly, a universal system for the expression of wild (i.e., not evolved) UPO genes in yeast has been recently claimed, including the production of first UPO from the ascomycete *Thermothelomyces thermophilus* (syn. *Myceliophthora thermophila*), among others [28,29]. Moreover, additional recombinant UPOs from the basidiomycete *Coprinopsis cinerea*, the ascomycete *Humicola insolens*, and other fungi have been independently produced in *Aspergillus oryzae (*by Novozymes, Bagsvaerd, Denmark) [25,30], and the above mentioned *Hsp*UPO [17], together with *Aspergillus niger* UPO, among others, [31] in *P. pastoris*.

These filamentous fungi and yeasts are the hosts of choice to scale-up UPO production for industrial application. However, *Escherichia coli* expression, as found in ~90% of all PDB entries, is the most widely used heterologous host for structure–function studies, where the lack of a glycosidic moiety (often variable) constitutes an advantage for protein crystallization and reproducible kinetic analyses. With this aim in mind, the already known *Mro*UPO and two new enzymes, from UPO-type genes of the ascomycetes *Collariella virescens* (r*Cvi*UPO) and *Daldinia caldariorum* (r*Dca*UPO), have been recently expressed in *E. coli* [32,33] and used in preliminary structure–function and other studies [34,35,36,37]. These and future-related investigations will strongly benefit from the results of the present study, where the crystal structures of the third and fourth fungal UPOs (after those from *A. aegerita* and *Hypoxylon* sp.) are reported and their different dimeric arrangements discussed.

## 2. Materials and Methods

### 2.1. Protein Expression and Purification

The production and purification of r*Mro*UPO and r*Cvi*UPO as active proteins in *E. coli* were reported by Carro et al. [34] and Linde et al. [33], respectively. In short, the amino-acid sequences of *Mro*UPO [32] and *Cvi*UPO [38] were converted into optimized nucleotide sequences using the Optimizer software [39], cloned into the pET23a plasmid, and transformed into *E. coli* BL21 cells, which were grown in an auto-induction medium [40] at 16 °C for 4 days to promote soluble protein production. After cell lysis and debris removal, the recombinant enzymes were purified by a combination of ion-exchange and size-exclusion chromatography (SEC) using an Äkta (GE Healthcare, Chicago IL, USA) fast liquid chromatography system. UPO fractions were identified by their absorbance around 420 nm, and activity was followed by 2,6-dimethoxyphenol (DMP) oxidation in the presence of H_2_O_2_, under the conditions described below.

In the case of r*Mro*UPO, the first purification step was on a 6 mL Resource™ Q cartridge using 10 mM phosphate (pH 7), supplemented with 1 M NaCl for elution. The appropriate fractions were pooled, concentrated, desalted, and loaded into a Mono Q high-resolution 5/5 column under the same conditions. Finally, the appropriate fractions were concentrated and, using 10 mM citrate (pH 4), dialyzed and loaded into a Mono S high-resolution 5/5 column and eluted under a NaCl gradient.

In the case of r*Cvi*UPO, the first step was cation exchange chromatography with a HiTrap SPFF column (GE Healthcare, Chicago, IL, USA) in 10 mM Tris (pH 7.4). The proteins, eluted with a gradient of the same buffer supplemented with 1 M NaCl (single peak with absorbance at 420 nm), were concentrated in an Amicon 3K device (Sigma-Aldrich, Saint Louis, MO, USA). The second step (to ensure purity) was SEC with a Superdex 75 column (10/300 GL; GE Healthcare, Chicago, IL, USA) in 10 mM Tris (pH 7.4) containing 0.15 M NaCl.

Enzyme purity was confirmed under denaturing conditions by 12% polyacrylamide gel electrophoresis (PAGE) in the presence of 0.1% sodium dodecyl sulfate (SDS) and 1% mercaptoethanol (5% in the loading buffer) [41]. Detection of the dimeric forms of the pure enzymes was performed also in 12% PAGE without any sample heating and using a loading buffer without any denaturing agent. Proper folding and binding of the cofactor were verified by analyzing the UV–visible spectrum of the enzymes (at the resting state) in 10 mM Tris (pH 7.4) using a Cary 60 spectrophotometer (Agilent, Santa Clara, CA, USA) (Figure S1, black profiles). Additionally, formation of the characteristic complex between reduced (ferrous) heme-thiolate enzymes (such as P450s and UPOs) and carbon monoxide (CO) was assessed in 0.2 M phosphate (pH 8) after addition of Na_2_S_2_O_4_ and CO flushing (Figure S1, red profiles). Concentrations were calculated using the r*Mro*UPO and r*Cvi*UPO molar extinction coefficients of ℇ_420_ 115.0 and 114.2 mM^−1^·cm^−1^, respectively.

### 2.2. Site-Directed Mutagenesis

To investigate *Mro*UPO dimerization through the intermolecular disulfide bridge visible in the crystal structure, the C227A mutation was introduced at its C-terminal tail using the Expand Long Template PCR kit from Roche (Basel, Switzerland) and the following primer (direct sequence with the mutated triplets underlined): 5′- CCA ACT GGC GAT AAC GCC GGC GCT ATC GTT CTC.

The above tail region was not solved in the r*Cvi*UPO crystal structure. However, to clarify its dimerization mechanism, (i) the single substitution C235A variant (changing the residue equivalent to *Mro*UPO Cys227 after sequence alignment) and (ii,iii) the tail deletion K228stop and P201stop variants (including the TAA stop codon) were generated using the above PCR kit and the following primers (direct sequences with the mutated triplets underlined): C235A, 5′- GGC AAA CAG GCA CCG CAA GCC C; K228stop, 5′- GCG TTA GTG ACC CCG GAA AAA CTG ATC GAC TAA; and P201stop, 5′- GGT TGG CGT CCG TAA AAA GCT GAA CTG.

The PCR products were digested with *Dpn*I and transformed into *E. coli* DH5α for propagation. Different r*Mro*UPO and r*Cvi*UPO variants were produced in *E. coli* as active cytosolic proteins and purified as described for the corresponding native enzymes.

### 2.3. Crystallization, Data Collection, and Processing

For the screening of crystallization conditions, the purified recombinant enzymes were extensively dialyzed at 4 °C against 10 mM Tris (pH 7.4), containing 0.15 M NaCl, and finally concentrated using Amicon 3K centrifugal filter units (Sigma Aldrich, Saint Louis, MO, USA) to 8–15 mg/mL. Crystals were grown by the sitting-drop vapor diffusion method at 22 °C using a Cartesian Honeybee X8 System (Genomic Solutions, Ann Arbor, MI, USA) in 96-well sitting-drop plates (Swissci MRC, Molecular Dimensions, Suffolk, England) with drops containing equal volumes (0.2 µL) of the concentrated protein solution and the reservoir solution equilibrated against 50 µL of the precipitant solution.

Specifically, crystals of r*Mro*UPO grew in 20% PEG 3000, 0.1 M sodium acetate, pH 4.5. Additionally, crystals of r*Cvi*UPO were obtained in 30% PEG 400, 0.1 M Tris, pH 8.5, 0.2 M sodium citrate. Crystals of the r*Cvi*UPO mutated variants were obtained in: (i) 25% PEG 5000 MME, 0.1 M Tris, pH 8.0, 0.2 M Li_2_SO_4_ for the C235A variant; and (ii) 20% PEG 6000, 0.1 M MES, pH 6.0, 30 mM MgCl_2_ for the K228stop variant. All crystals were soaked in cryoprotectant solutions containing the mother liquor supplemented with 30% (*v*/*v*) PEG 400 and flash-cooled in liquid N_2_ prior to data acquisition.

Diffraction data were collected at the BL13-XALOC beamline of the ALBA Synchrotron (Cerdanyola del Vallés, Barcelona, Spain). Crystallographic data were processed using XDS [42] and merged and scaled with AIMLESS [43]. Details are shown in Table 1.

### 2.4. Structure Determination and Refinement

The structures were solved by molecular replacement. The r*Mro*UPO structure was solved with the program PHASER [44] implemented in the CCP4 package, using the crystal structure (2YOR) of *Aae*UPO [14] as the search model. Structural refinement of the initial model was carried out using Refmac [45] alternating manual building, addition of water molecules, and positioning of the heme group and ligands using Coot [46]. Once the structure of r*Mro*UPO was solved, the structural model was used to determine the structure of r*Cvi*UPO and its C235A and K228stop variants. Structural refinement of the latter enzyme and variants used the same workflow as the applied for r*Mro*UPO. Details of the model refinements are given in Table 2.

The coordinates and structure factors have been deposited in the Protein Data Bank (as entries 7ZBP and 7ZCL). Figures for structural representations were drawn with the Pymol [47] and Swiss PdbViewer [48] programs. Protein–protein interactions were analyzed with PISA [49] available at EMBL-EI (PDBePISA; http://www.ebi.ac.uk/msd-srv/prot_int/pistart.html, accessed on 1 March 2022). The final molecular models were analyzed and validated with MolProbity [50]. The nonresolved C-terminal tail was tentatively modeled with AlphaFold [51] at ColabFold (https://colab.research.google.com, accessed on 1 March 2022) [52].

### 2.5. Size-Exclusion Chromatography and Analytical Ultracentrifugation

The UPO molecular masses at the native as well as at the unfolded and disulfide-free states were first estimated as described at the end of the purification section by using (i) Superdex-75 SEC and (ii) SDS-PAGE under denaturing and nondenaturing conditions (with and without heating at 100 °C with SDS and mercaptoethanol, respectively). Molecular-mass standards were used in both cases: (i) conalbumin (75 kDa), ovalbumin (43 kDa), carbonic anhydrase (29 kDa), ribonuclease A (13.7 kDa), and aprotinin (6.5 kDa) for SEC; and (ii) the Precision Plus protein standards (10–250 kDa) from Bio-Rad (Hercules, CA, USA) for SDS-PAGE.

Moreover, analytical ultracentrifugation was used in sedimentation velocity experiments with native r*Cvi*UPO and two mutated variants. These experiments were performed at the Molecular Interactions facility of CIB using a Beckman-Coulter (Brea, CA, USA) analytical ultracentrifuge Optima XLI. The sedimentation coefficient distributions were calculated by least-square boundary modeling of sedimentation velocity data using the continuous distribution *c(s)* Lamm equation mode [53], as implemented in SEDFIT 16 1c, with a confidence level of 0.68.

In parallel to the above sedimentation velocity experiments, dynamic light scattering (DLS) experiments were performed in a Protein Solutions (Piscataway, NJ, USA) DynaPro MS/X instrument, using a 90° light scattering cuvette. Data were collected and analyzed with the Dynamics V6 software yielding the corresponding diffusion coefficients (*D*) that, together with the sedimentation coefficients (*s*), were used to establish the molecular mass (*M*) of the enzymes using the Svedberg equation, Equation (1), where *T*, *R*, ῡ, and ρ are the absolute temperature, the universal gas constant, the partial specific volume of the protein, and the density of the solution, respectively.

(1) $$M=\frac{RTs}{\left(1-ῡ\rho \right)D\;}$$

### 2.6. Enzyme Kinetics

First, the optimal pH for the oxidation of three UPO reducing substrates—veratryl alcohol (10 mM), 2,2′-azino-bis(3-ethylbenzothiazoline-6-sulfonic acid) (ABTS, 2 mM) and DMP (15 mM)—by each enzyme was analyzed at pH 2–10 in 0.2 M Britton–Robinson buffer, at 24 °C, using 1 mM H_2_O_2_.

Then, kinetic curves for the above enzyme-reducing substrates were obtained from the initial (10–30 s) increase in absorbance due to product formation, using a Thermo (Waltham, MA, USA) Spectronic spectrophotometer at the optimal pH values. Reactions included oxidation of 0.09–10 mM veratryl alcohol in 0.1 M acetate, pH 4.6 for r*Mro*UPO and pH 3 or 5 for r*Cvi*UPO (veratraldehyde ℇ_310_ = 9300 M^−1^·cm^−1^), 0.04–50 mM ABTS in 0.1 M acetate, pH 5 for r*Mro*UPO and pH 4 for r*Cvi*UPO (ABTS cation radical ℇ_436_ = 29,300 M^−1^·cm^−1^), and 0.03–30 mM DMP in 0.1 M tartrate, pH 4 for r*Mro*UPO and pH 5 for r*Cvi*UPO (ℇ_469_ = 29,300 M^1^·cm^1^). The reactions (1 mL) were triggered by the addition of 5 mM H_2_O_2_ for r*Mro*UPO or 1 mM H_2_O_2_ for r*Cvi*UPO_._

Kinetic curves for the enzyme-oxidizing H_2_O_2_ substrate were obtained with 0.5 to 30 mM H_2_O_2_ in 0.1 M acetate (pH 4) containing 7.5 mM DMP, whose (one electron) oxidation was monitored for activity estimation, and the H_2_O_2_ constants were obtained (note that two moles of reducing substrate are oxidized by each mole of peroxide).

Curve fitting and data analysis for kinetic constant estimation (from triplicate reactions) were carried out with Sigma Plot 11.0. Michaelis–Menten constant (*K*_m_), turnover number (catalytic constant, *k*_cat_), catalytic efficiency (*k*_cat_/*K*_m_), and their standard errors were generally obtained by nonlinear fitting the *k*_obs_ values to Equation (2) (Michaelis–Menten model).

(2) $$f=\frac{k_{\mathrm{cat}\;}S}{K_{m}+S}$$

However, Equation (3) was used for r*Cvi*UPO kinetics with ABTS and H_2_O_2_, where enzyme inhibition was observed (with the *k*_i_ inhibition constant being the concentration producing half of the maximal inhibition).

(3) $$f=\frac{k_{\mathrm{cat}}}{1+\frac{K_{m}}{S}+\frac{S}{k_{i}}}$$

### 2.7. Fatty Acid Enzymatic Oxygenation

For evaluating the UPO oxygenation (epoxidation vs. hydroxylation) ability, the following 18-C unsaturated fatty acids (from Sigma-Aldrich, Saint Louis, MO, USA) were used as substrates: oleic (*cis*-9-octadecenoic, C18:1), linoleic (*cis*,*cis*-9,12-octadecadienoic, C18:2), and α-linolenic (*cis,cis,cis-*9,12,15-octadecatrienoic, C18:3). Thirty-minute reactions were performed using 0.1 mM substrate, 0.25 or 0.40 µM enzyme (in the C18:1/C18:3 and C18:2 reactions, respectively), and 1.25 mM H_2_O_2_ in 50 mM phosphate (pH 7) at 30 °C in the presence of 20% acetone (for better fatty acid solubility).

Products (and remaining substrates) were liquid–liquid extracted with methyl *tert*-butyl ether (Sigma-Aldrich, Saint Louis, MO, USA), which was evaporated under a N_2_ stream. *N*,*O*-Bis(trimethylsilyl)trifluoroacetamide (Supelco, Bellefonte, PA, USA) was used to prepare trimethylsilyl derivatives. Analyses were performed with an Agilent (Santa Clara, CA, USA) gas chromatography–mass spectrometry (GC–MS) QP2010 Ultra equipment using a fused-silica DB-5HT 30 m capillary column from J&W Scientific (Folsom, CA, USA). The oven was heated from 120 °C (1 min) to 300 °C (15 min) at 5 °C min^−1^. The injector and transfer line were kept at 300 °C. Compounds were identified by mass fragmentography and comparison of their mass spectra with authentic standards. Quantifications were obtained from total-ion peak areas (partially overlapping peaks were deconvoluted when required) using external standard curves and molar response factors of the same or similar compounds.

## 3. Results

### 3.1. Production of Native UPOs and Site-Directed Variants

The nonmutated recombinant (hereinafter native) r*Mro*UPO and r*Cvi*UPO were obtained as soluble and active proteins in *E. coli*, purified without any tags, and used for crystallization, analytical ultracentrifugation, and catalytic studies. These studies were often favored by the higher expression levels and easier purification of the r*Cvi*UPO enzyme.

Moreover, to confirm some of the results obtained, the r*Mro*UPO C227A and r*Cvi*UPO C235A, K228stop, and P201stop variants were generated by site-directed mutagenesis, isolated by ion-exchange chromatography and SEC, and characterized by biophysical and crystallographic methods, as done with the native enzymes, except for the P201stop variant whose extremely low expression levels prevented isolation with significant yields.

The results obtained in the above studies are described in the next five sections. For consistency with residue numbering in wild enzymes (mature proteins isolated from fungal cultures), the signal sequence and introduced initial methionine were not considered for residue numbering in recombinant UPOs (r*Mro*UPO and r*Cvi*UPO included) except for *Hsp*UPO [17].

### 3.2. Overall Crystallographic Structures

Crystals of recombinant r*Mro*UPO and r*Cvi*UPO were obtained at sufficiently high resolution (1.45 and 1.95 Å, respectively), and the corresponding structures solved by molecular replacement. The r*Cvi*UPO C235A and K228stop variants were also crystallized, but the molecular structures obtained (not shown) were fully superimposable with that of the native (nonmutated) enzyme except for the residue(s) substituted or removed.

**Figure 1:**
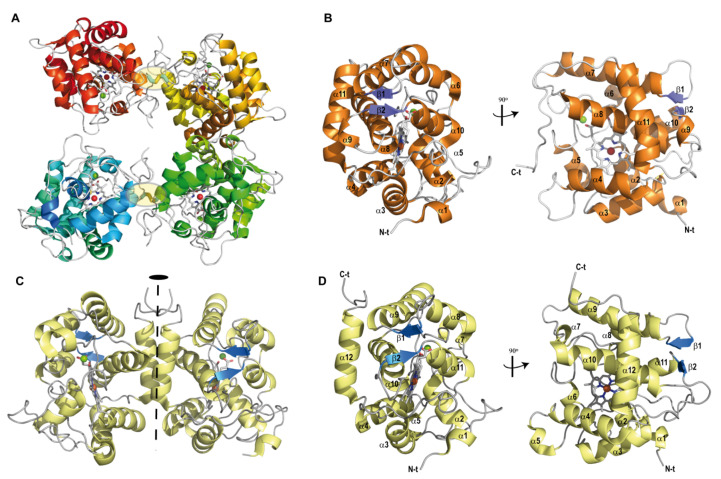
Ribbon diagrams of UPO crystal structures. (**A**) Asymmetric unit of r*Mro*UPO (7ZBP). Each of the two homo-dimers is stabilized by a disulfide bridge (C227-C227′, highlighted in yellow) across the subunit interface, and dimers further assemble into a tetramer. (**B**) Overall structure of compact monomeric r*Mro*UPO containing 11 α-helices (orange) and two short antiparallel β-strands (blue). For clarity, a 90° rotation to show the catalytic pocket is included. (**C**) Crystallographic structure of r*Cvi*UPO (7ZCL) showing a compact dimer in the asymmetric unit. Dimerization occurs through a parallel association of α-helices (α12) mainly stabilized by hydrophobic interactions (the two-fold axis is shown by a dashed line). (**D**) The monomeric r*Cvi*UPO structure contains 12 α-helices (yellow) and two short antiparallel β-strands (blue). As in (**B**), a 90°rotation is shown in (**D**). One heme cofactor (CPK sticks with Fe^3+^ as a red sphere) and one Mg^2+^ ion (green sphere) are also present in each UPO monomer.

**Figure 2:**
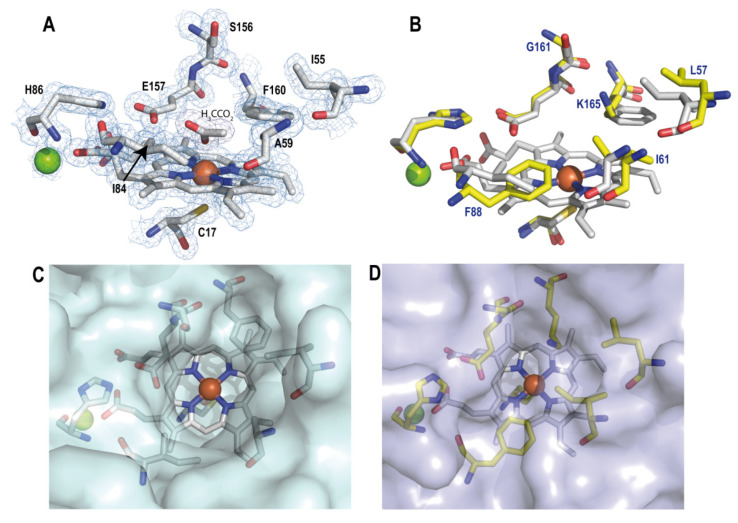
Heme pocket residues (top) and access channels (bottom). (**A**) View of the heme cavity in r*Mro*UPO with Ile84, His86, Glu157, and Phe160 at the upper side and the characteristic proximal Cys17 at the lower side (neighbor I55, A59, and S156 residues and acetate ion are also shown). The 1.4 Å resolution 2Fo-Fc electron density map (blue) contoured at 1.0 σ is included. (**B**) Active site differences between r*Mro*UPO (gray) and r*Cvi*UPO (yellow). Only noncommon residues in r*Cvi*UPO, including Leu57, Ile61, Phe88, Gly161, and Lys165, are labeled. (**C**,**D**) Semitransparent surfaces show the access channel to the heme in r*Mro*UPO and r*Cvi*UPO, respectively. The equivalent positions of Ala59, Ile84, and Phe160 in r*Mro*UPO change to Ile61, Phe88, and Lys165 in r*Cvi*UPO, reducing the entrance to the channel. These residues and acetate ion are shown as CPK sticks, while the Fe^3+^ and Mg^2+^ ions are shown as red and green spheres, respectively.

The asymmetric unit of the r*Mro*UPO crystal structure (Figure 1A) includes four polypeptide chains that associate to form a tetramer composed of two homo-dimers in a head-to-tail arrangement, each of them stabilized by an intermolecular disulfide bridge (between the Cys227 residue of both molecules). Each of the r*Mro*UPO monomers (Figure 1B) is compact and spherical in shape and consists of residues from Ser1 to Glu235, which displayed clearly defined electron density (only lacking the C-terminal Leu236), together with one heme molecule and one Mg^2+^ ion. The overall fold is mainly helical with eleven α-helices and two short β-strands (V120-N122 and E174-P176). Moreover, a clear electron density could be detected at the active site (Figure 2A) that could be unequivocally assigned to an acetate ion from the crystallization medium. Both active sites in the dimer are open and accessible from the solvent (see below).

The r*Cvi*UPO asymmetric unit reveals a compact dimer through a parallel association of helix α12 from each monomer, providing a closely packed helix interface (Figure 1C). The r*Cvi*UPO structure includes all amino acid residues, except for the initial Glu1 and the thirty-two C-terminal residues—from Lys228 to Lys259, whose lack is due to their flexible conformation—one heme molecule and one Mg^2+^ ion. The monomer adopts an α fold (Figure 1D) formed by twelve helices and two short β strands (E124-V126 and K177-P179). The α-helices include two short α5 and α7 helices not found in r*Mro*UPO, whereas the short α9 helix present in r*Mro*UPO is absent from this structure. In spite of the analogies, a noticeable feature was the different dimer association, in which no intermolecular disulfide bridge was observed. Instead, the abovementioned parallel association between two α-helices at the dimer interface is proposed to play a key role.

### 3.3. Heme Pocket

The stereochemical quality of the above crystal models is illustrated with the unambiguous electron density map at 1.45 Å resolution, shown in Figure 2A. The map corresponds to the heme cofactor, located at a central position in the crystal structure of r*Mro*UPO (Figure 1B), its surrounding residues, and the acetate and Mg^2+^ ions.

The heme cofactor sits in an internal pocket with its iron ion coordinated by the sulfur atom of r*Mro*UPO Cys17 at a distance of 2.4 Å in the lower (also known as proximal because of the cysteine ligand) side of the heme cofactor. The heme pocket includes residues lining the upper (also known as distal) side of the cavity, such as His86, Ile84, Phe160, Glu157, Ser156, Ile55, and Ala59, among others (Figure 2A). One of the acetate oxygens is near the sixth coordination position of the heme iron. The Mg^2+^ ion appears coordinated (at 2.07–2.19 Å distances) by the Ser89 hydroxyl, the His86 (backbone) carbonyl, two water molecules (wat1 and wat2), and the carboxylates of Glu85 and heme ring-D propionate (neighbor Asp87 is not involved in Mg^2+^ coordination). In this way, the Mg^2+^ cation would contribute to anchoring the heme cofactor inside the UPO central pocket.

In r*Cvi*UPO, the neighbor residues located at the upper side of the cofactor that outline the cavity shell include Leu57, Ile61, His90, Phe88, Gly161, Glu162, and Lys165, with the heme iron coordinated by the thiol side chain of Cys19 at the lower side (Figure 2B). The Mg^2+^-coordinating residues in r*Cvi*UPO (at 2.01–2.14 Å) are the same found in r*Mro*UPO, but no acetate was found at the heme pocket. This is in agreement with its absence from the r*Cvi*UPO crystallization medium, and resulted in the presence of one water molecule (wat255) near the sixth coordination position of the heme iron.

Three of the above residues are invariable in the two enzymes (Figure 2A,B): (i) Cys17/Cys19, acting as the fifth ligand of the heme iron (together with the four N atoms of the heme macrocycle); and (ii) Glu157/Glu162 and His86/His90, putatively contributing to the reaction with H_2_O_2_ for heme activation (the conserved histidine also contributes to the coordination of the Mg^2+^ ion through its backbone carbonyl). Concerning the other five residues (Figure 2A,B), it is interesting to mention that a phenylalanine was present in both r*Mro*UPO and r*Cvi*UPO structures, although it occupies opposite positions (Phe160 and Phe88, respectively). Phe160 of r*Mro*UPO was replaced by Lys165 in r*Cvi*UPO and, as a result of this and other amino acid substitutions, the access to the substrate binding site in r*Cvi*UPO was narrowed (Figure 2C,D).

### 3.4. Dimeric Arrangements

**Figure 3:**
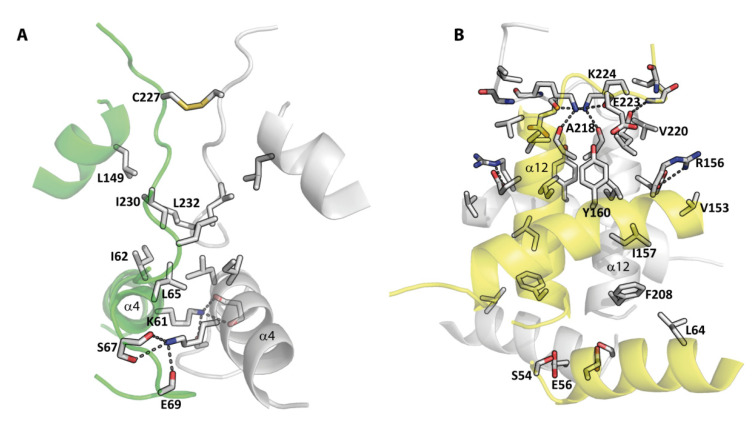
Schematic representation of the dimer interfaces in r*Mro*UPO (**A**) and r*Cvi*UPO (**B**). Subunits of the dimers are shown in gray and green for r*Mro*UPO, and gray and yellow for r*Cvi*UPO. Hydrogen bonds and salt bridges together with hydrophobic interactions contribute to the stability of the dimers, together with the C227-C227′ disulfide bridge and the parallel association of the two α12 helices (also see Figure 1).

As explained above, the r*Mro*UPO and r*Cvi*UPO crystal structures revealed dimeric proteins in both cases, but suggested the existence of two different dimeric arrangements. As already depicted in Figure 1A,C, an intermolecular disulfide bridge and the association between parallel α-helices appeared as main dimerization motifs in the r*Mro*UPO and r*Cvi*UPO crystal structures, respectively. However, other interactions are also produced at the dimer interfaces of the two enzymes (Figure 3A,B), most probably contributing to the whole dimeric arrangement.

In r*Mro*UPO, the dimer interface buries 825 Å^2^ of each monomer and is formed from the interactions between residues of the C-terminal loop and residues from the helix α4 (Figure 3A). From the C-terminal region, an intermolecular disulfide bond between the sulfhydryl side chains of Cys227 and the hydrophobic interactions established by Ile230 and Leu232 contribute to the clustering of the dimer. In addition, residues Ile62-Glu69 from the helix α4 increase the dimer stability by polar interactions of Lys61 with Ser67 and Glu69, and by a patch of hydrophobic residues at the interface (Ile62, Leu65).

In the r*Cvi*UPO crystal structure (Figure 3B), the buried interface area of the dimer, including the two α12 helices, was 1304 Å^2^. Dominant at the upper side of the interface are Arg156 and Lys224, which establish polar contacts on both sides with residues Glu216 and Glu223, respectively. At the corner of the dimer, Ser54 and Glu56 also bridge the interface by hydrogen bond interactions.

PDBePISA analyses were employed to unveil the interface interactions between monomers of the native and mutated enzymes. The dimeric model built with AlphaFold for the whole C227A variant of r*Mro*UPO revealed a contribution of hydrophobic interactions to the homo-dimer formation, with an estimated free energy of −18.8 kcal/mol. This value was similar to that produced in the crystallographic dimer of native r*Mro*UPO (−20.1 kcal/mol). In fact, even lower differences were found when the C227A model was compared with an AlphaFold model for the whole r*Mro*UPO, with −18.3 kcal/mol free energy. This supports that, in addition to the disulfide bond contribution to dimeric arrangement, hydrophobic interactions also play an important role in the formation of a r*Mro*UPO stable dimer (even in the absence of Cys227).

In the case of r*Cvi*UPO, the PDBePISA analyses of the AlphaFold models of C236A, K228stop, and P201stop showed lower numbers of H bonds (four, two, and one, respectively) compared to the whole (AlphaFold model) native r*Cvi*UPO (with nine H bonds). Moreover, the hydrophobic interactions in the C236A and K228stop interface (between −24.0 and −24.3 kcal/mol free energies) were similar to those found in the whole native r*Cvi*UPO. However, this interaction strongly decreased in the P201stop model (with a free energy of −16.9 kcal/mol), confirming the central contribution of such parallel association between the α12 helices of the two monomers, being absent from the P201stop variant.

### 3.5. Biophysical Properties: Molecular-Mass and Oligomerization State

**Figure 4:**
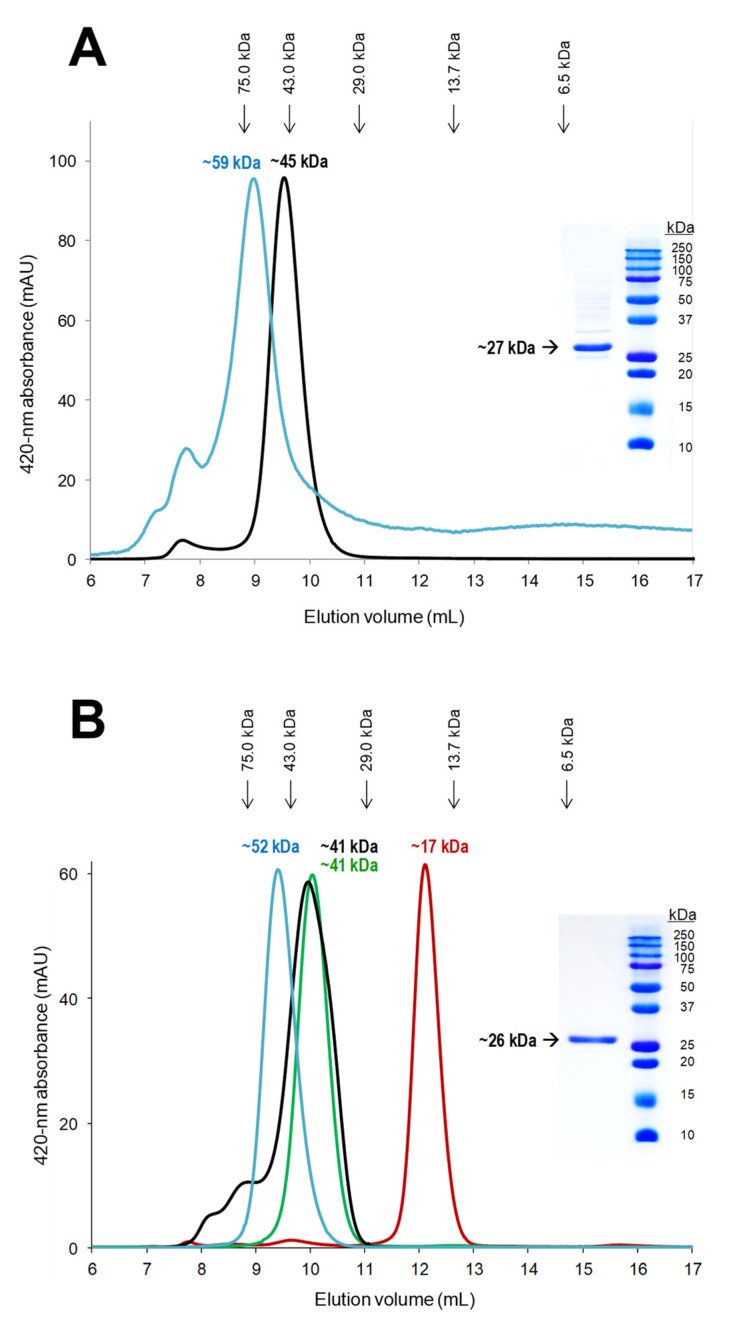
SEC profiles and SDS-PAGE images (insets) of recombinant UPOs and variants (the positions of standards are indicated). (**A**) r*Mro*UPO (black) and its C227A variant (blue). (**B**) r*Cvi*UPO (black) and its C235A (blue), K228stop (green), and P201stop (red) variants. SEC was performed with a Superdex 75 column and isocratic elution with 10 mM Tris (pH 7.4) containing 0.15 M NaCl (given the low production of the C227A variant, its absorbance in A is multiplied by a factor of 15). SDS-PAGE was performed with 0.1% SDS containing 1% mercaptoethanol, and same protein amount (~1.7 µg) was loaded in each case.

Firstly, the molecular masses of r*Mro*UPO and r*Cvi*UPO (under native conditions) were estimated by SEC after enzyme purification or during the last purification step (when purification concluded with SEC and the expression level was too low, as for the P201stop variant of r*Cvi*UPO). These results were compared with the molecular masses estimated after protein unfolding (also resulting in cofactor release) and breakdown of disulfide bridges, by SDS-PAGE in the presence of mercaptoethanol. As shown in Figure 4 (black profiles) and insets, the SEC molecular masses (41–45 kDa) of the native UPOs were nearly two-fold the masses from SDS-PAGE (26–27 kDa), which in turn were similar to the theoretical masses estimated from the protein sequences (23–30 kDa). Monomeric and dimeric forms of both enzymes could also be observed by SDS-PAGE with and without heating at 100 °C with SDS and mercaptoethanol, respectively (Figure S2). These results indicate that both r*Mro*UPO and r*Cvi*UPO are dimeric proteins under physiological conditions.

Molecular masses (from SEC) similar to or even higher than those found for the native enzyme were obtained for the C227A variant (~59 kDa) of r*Mro*UPO (Figure 4A, blue profile) and the first two r*Cvi*UPO variants analyzed—K228stop (~41 kDa) and C235A (~52 kDa) that present 32-residues shorter C-terminal tail and the Cys→Ala mutation, respectively (Figure 4B, green and blue profiles, respectively)—suggesting that their oligomerization state was not modified. However, the SEC analysis of the P201stop variant, with a 27-residue shorter C-terminal tail than K228stop (and 59-residue shorter than native r*Cvi*UPO), provided a mass value of only ~17 kDa (indicative of a monomeric protein), revealing that these additional 27 residues removed were strongly involved in dimerization.

**Figure 5:**
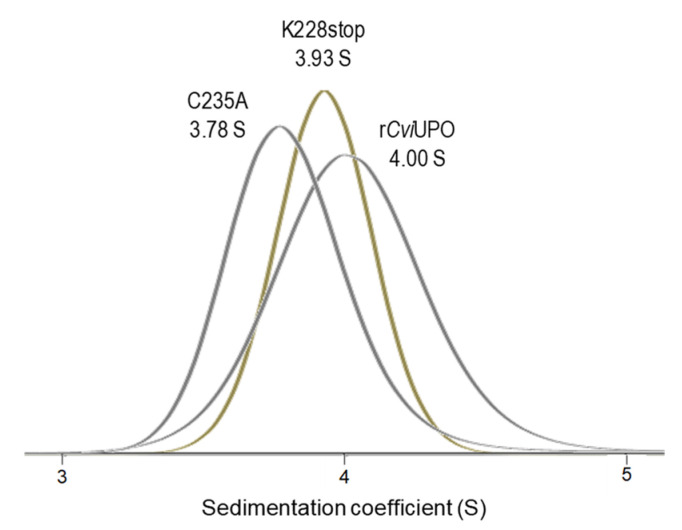
Sedimentation coefficients of native r*Cvi*UPO and its K228stop and C235A variants (from sedimentation velocity profiles in Figure S3). The estimated molecular masses were in the range of 57–66 kDa, revealing dimeric proteins in all the cases.

To confirm the above SEC analyses of r*Cvi*UPO and variants, analytical ultracentrifugation was performed and the sedimentation velocity profiles (Figure S3 left) and *c(s)* distributions (Figure S3 right) were obtained for the native enzyme and its C235A and K228stop variants (not enough P201stop enzyme was available, as explained above). In the sedimentation velocity assays, sedimentation coefficients of the major native r*Cvi*UPO, C235A, and K228stop species (representing 95.1, 94.6, and 98.7%, respectively) were determined to be 4.00, 3.78, and 3.93 S (Figure 5).

With the above S values and the diffusion coefficients from parallel DLS analyses, molecular masses of 60 (r*Cvi*UPO), 66 (C235A), and 57 (K228stop) kDa were estimated using the Svedberg equation. The slightly lower S values and higher masses estimated for the two variants lacking (i) only Cys235 (substituted by an alanine residue) and (ii) Cys235 and other 31 residues in a shortened C tail (K228stop) could be due to a modification of the hydrodynamic (and sedimentation) properties of the enzyme by the breakdown of the intramolecular disulfide bridge in the mobile C tail region predicted by AlphaFold (Figure S4), which would be similar to that stabilizing the C-terminal tail of *Aae*UPO [14]. In any case, the results support the SEC analysis confirming that r*Cvi*UPO remains a dimer in the absence of Cys235.

### 3.6. Comparison of Catalytic Properties

To investigate if the dimeric arrangements would affect the catalytic properties of the two short UPOs under investigation, the kinetic constants of the native enzymes and some of the mutated variants on four peroxygenase/peroxidase classical substrates were estimated (Table 3). First, the constants for H_2_O_2_ were determined using DMP as enzyme-reducing substrate. It was observed that r*Cvi*UPO was inhibited by high H_2_O_2_ concentrations (with *k*_i_ of 1.1 mM). Therefore, the r*Cvi*UPO and r*Mro*UPO catalytic constants (towards veratryl alcohol, ABTS and DMP) were determined at 1 mM and 5 mM H_2_O_2_ concentrations, respectively.

In the case of r*Mro*UPO, a comparison with the activities reported for the wild enzyme isolated from *M. rotula* cultures [22], confirmed that the recombinant enzyme is able to oxidize the same substrates and to reduce H_2_O_2_ with catalytic efficiencies in a similar order of magnitude. However, removal of the intermolecular disulfide bridge observed in the crystal structure by replacement of Cys227 by alanine caused complete loss of the enzyme activity, in spite of the fact that the enzyme maintains its dimeric nature, as explained above.

Comparison with the wild *Cvi*UPO could not be performed, since this UPO is only known as a recombinant enzyme. Concerning the two recombinant enzymes, r*Cvi*UPO was over 60-fold less efficient oxidizing veratryl alcohol than r*Mro*UPO (due to lower substrate affinity) but over 10-fold more efficient oxidizing ABTS (due to higher turnover) revealing marked substrate preferences. Moreover, in contrast to activity loss by the C227A mutation in r*Mro*UPO, no decrease in the catalytic efficiency was produced by the C235A mutation in r*Cvi*UPO. Interestingly, strong changes in both veratryl alcohol activity (25-fold higher turnover) and affinity (19-fold higher *K*_m_) were caused by the K118stop mutation (although the resulting efficiency was only slightly increased).

Finally, the oxygenation patterns by native r*Cvi*UPO and its C-terminal tail variants were studied on 18-C unsaturated fatty acids. In all cases, the conversion yields lay in the 50–98% range and (different) epoxides were found as the main products. The most interesting chromatographic profile was observed for the linoleic acid reaction with the above K228stop variant (Figure S5). As shown by the quantitative results provided in Table 4, the variant and native enzyme produced similarly high (97–98%) conversions, but over 50% of the products formed by K228stop were diepoxides, which are of interest as cross-linking molecules, while they were fully absent from the r*Cvi*UPO reactions, where monoepoxides predominate. This higher diepoxide content resulted in up to 68% epoxidation yield (defined as the percentage of total double bonds converted into epoxides) by the variant with shortened tail.

## 4. Discussion

Novel structural–functional information on short UPO enzymes is provided by a combination of crystallographic, biophysical, and molecular biology techniques, with the focus on the dimerization mechanisms. For these studies, we took advantage of the heterologous production of r*Mro*UPO and r*Cvi*UPO in *E. coli* as soluble enzymes [33,34], which strongly facilitate crystallization by reducing the heterogeneity due to the glycosidic moiety, as shown for in vitro deglycosylated yeast-expressed recombinant UPOs (with 30–50% carbohydrate content) [14,15,17]. Moreover, it has been shown that the activity and stability properties of the r*Mro*UPO obtained are fairly similar to those of wild *Mro*UPO isolated from fungal cultures [54].

### 4.1. Short and Long UPO Families

Phylogenetic analyses revealed two major UPO families, characterized by their shorter or longer protein chains, each family including several thousand sequences in different subfamilies [12]. The long *Aae*UPO is the best known UPO in terms of crystal structure [14], directed evolution for yeast expression [27], and reactions of biotechnological interest [4,55], among other studies. While long UPOs are only present in higher fungi, short UPOs have a wider distribution and more ancient origin inside the fungal kingdom [12].

**Figure 6:**
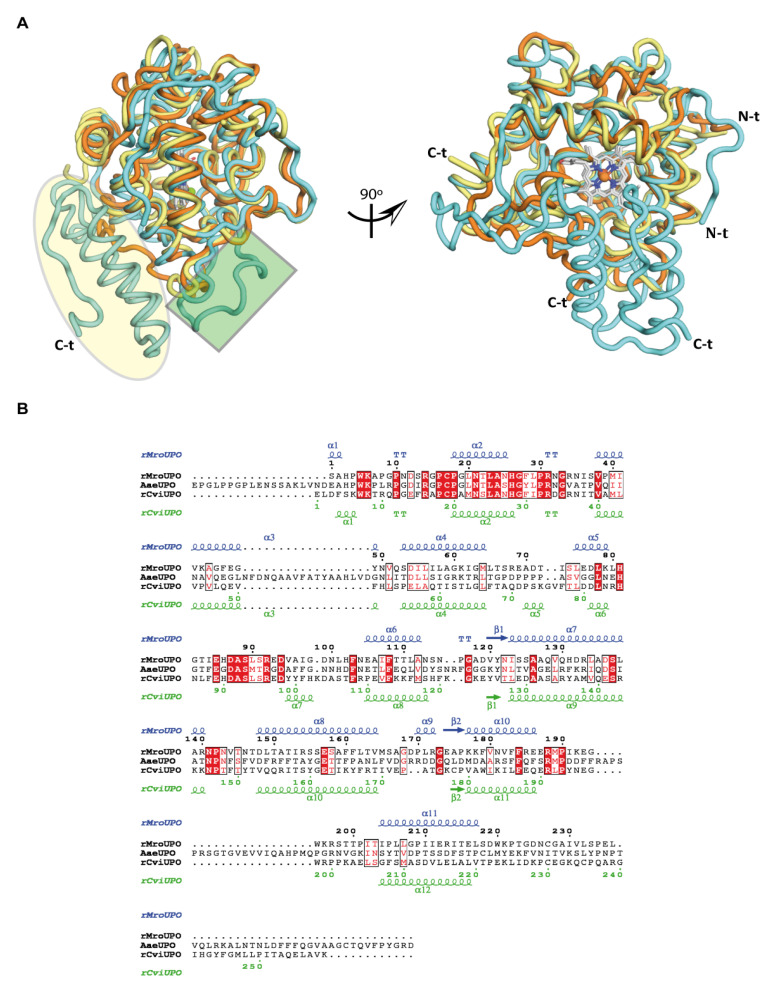
Structural and sequence comparisons. (**A**) Structural alignment of r*Mro*UPO (7ZBP, orange), r*Cvi*UPO (7ZCL, yellow), and *Aae*UPO (2YOR, cyan). The main differences are at the long loop in *Aae*UPO in relation to the loop including the α3 helix in the short UPOs (green square), and the longer C-terminal tail in *Aae*UPO (highlighted in yellow). (**B**) Sequence alignment of the two short UPOs with *Aae*UPO. Strictly conserved residues are shown in red boxes, and red characters in white boxes specify similarity. The top and bottom lanes show the secondary structure of r*Mro*UPO and r*Cvi*UPO, respectively, with α, β, and TT representing α helices, β sheets, and turns, respectively.

Structural and sequence differences are illustrated in Figure 6, where *Mro*UPO and *Cvi*UPO were compared with the model *Aae*UPO. The two main structural characteristics of the latter long UPO with respect to the two short UPOs (Figure 6A left) concern its (i) 18-residues long internal loop, and (ii) longer C-terminal tail with respect to short UPOs (the *Aae*UPO C-terminal tail is >50-residues longer than the r*Mro*UPO tail and >30-residues longer than the r*Cvi*UPO tail). *Aae*UPO also has a near 17–19 residues longer N-terminal tail with respect to the two short UPOs (Figure 6A right). Significant similarities and differences between the two UPO families are also found at the active-site level, as discussed below. The former include the conserved proximal cysteine and distal glutamic acid, indicated with a red background in Figure 6B, together with other conserved residues that, interestingly, are especially frequent in the N-terminal moiety of these proteins (which harbors 74% of the conserved residues).

Concerning their active sites, all UPOs from basidiomycetes—such as *Aae*UPO, *Cci*UPO, and *Mro*UPO—and ascomycetes—such as *Hsp*UPO, *Dca*UPO, *Cgl*UPO, *Hin*UPO, and *Cvi*UPO—whose molecular structure has been modeled [14,17,33,56,57,58,59] (Figure S6), show two invariable cysteines (Cys19 in *Cvi*UPO) and glutamic acid (Glu162 in *Cvi*UPO) residues at the lower and upper sides of the heme pocket. These conserved residues would (i) act as the fifth ligand of the heme iron, and (ii) contribute to the reaction with H_2_O_2_, respectively. A water oxygen is often near the sixth coordination position of the heme iron in the resting enzyme (as shown in Figure S6H for r*Cvi*UPO) that is substituted by the oxygen of acetate, when present in the crystallization medium (as shown in Figure S6G for r*Mro*UPO), or by O or N atoms of other buffers or ligands as described for *Hsp*UPO [17] and evolved *Aae*UPO [15]. A second residue would contribute to the activation of UPOs by H_2_O_2_, being an arginine (Arg189) in the long *Aae*UPO and *Cci*UPO (Figure S6A,B), and a histidine (His90 in *Cvi*UPO) in the other (short) UPOs mentioned above (Figure S6C–H). These two residues act as an acid–base pair in compound I formation through a mechanism [60] shared by peroxidases and peroxygenases.

Several aromatic residues are also present at the UPO heme pocket and most probably modulate the access of different substrates to the reactive iron-oxo group of the peroxide-activated heme, as suggested for r*Aae*UPO [15]. These residues include: (i) *Aae*UPO/*Cci*UPO Phe121 near the heme edge, conserved in *Cvi*UPO (as Phe88) but substituted by isoleucine (Ile84 in *Mro*UPO) or leucine (in the other four UPOs mentioned above); and (ii) *Aae*UPO/*Cci*UPO Phe199, with one tyrosine (Tyr166 in *Cvi*UPO), one phenylalanine (Phe160 in *Mro*UPO), or both tyrosine and phenylalanine residues (in the other four UPOs) occupying neighbor (but not homologous) positions at the upper side of heme. *Aae*UPO Phe199 and *Hsp*UPO Phe176 have been reported to interact with aromatic substrates [14,15,17]. As a result of differences in the heme pocket and neighboring residues, the size of the access channel varies in the different UPO crystal structures, with the internal diameter being in the order r*Mro*UPO > r*Hsp*UPO > r*Cvi*UPO > *Aae*UPO (Figure S7A–C, respectively).

### 4.2. Two Different Dimeric Arrangements

Long UPOs are only known as monomeric enzymes, while dimerization has been reported in short UPOs, such as *Mro*UPO, *Mwe*UPO [23], and *Cvi*UPO [33], although not for short *Hsp*UPO [17]. The first experimental evidence for dimer formation by r*Mro*UPO and r*Cvi*UPO came from double molecular-mass estimation by techniques potentially preserving or destroying (by the action of SDS and mercaptoethanol) the tertiary and quaternary structure of the native UPOs. The results obtained, a molecular mass reduction from 40–45 to 26–27 kDa, clearly showed that both r*Mro*UPO and r*Cvi*UPO are dimeric enzymes.

Analytical ultracentrifugation is a powerful technique to study protein associations under physiological (ecological) conditions [61] and, in combination with dynamic light scattering, enabled more precise estimation of the molecular masses (57–66 kDa) of the two native UPOs and their mutated variants, although the molecular mass of the P201stop variant could only be estimated by SEC, due to its very low expression level as an active soluble enzyme in *E. coli*.

**Figure 7:**
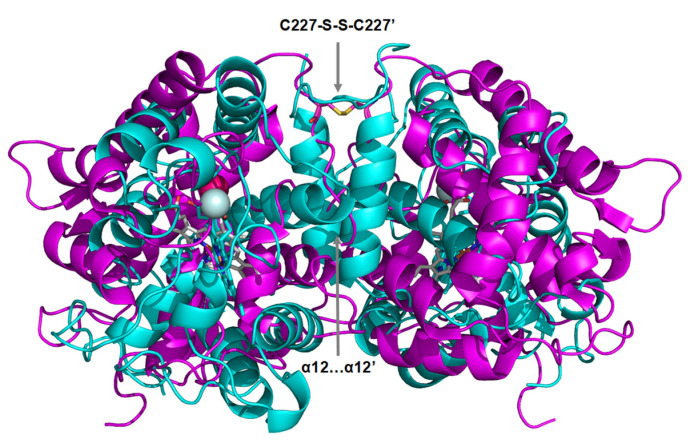
Two dimeric arrangements in short UPOs, as shown by the superimposition of the r*Mro*UPO (purple) and r*Cvi*UPO (cyan) crystal structures. The interface of the dimeric ribbon models shows parallel association of two α–helices in r*Cvi*UPO and an intermolecular disulfide bridge in r*Mro*UPO. Heme cofactors are shown as CPK-colored sticks and Mg^2+^ ions as spheres.

Unexpectedly, considering the proposed dimerization of *Mro*UPO by an intermolecular disulfide bridge [1,57] based on the unpublished 5FUJ/5FUK coordinates of the wild enzyme available at PDB (which are superimposable with those of the recombinant enzyme, 7ZBP, solved here), the C227A variant still appeared as a dimer. The same result was found, using both SEC and analytical centrifugation, by the C235A mutation in r*Cvi*UPO. The solved crystal structures explained this situation, and agree with a dimeric form in the solution state. First, both asymmetric units showed dimeric structures (associated in a tetramer in the case of r*Mro*UPO crystals) confirming the biophysical analyses. Moreover, a strong parallel association between two α-helices was observed at the interface of the r*Cvi*UPO dimer. Its removal in the shortened P201stop variant strongly reduced the molecular mass, demonstrating a main role in the dimerization of this enzyme. The extremely low expression and difficult purification of this variant, mentioned above, could be related to lower stability of the monomeric form, as claimed for some dimeric hemeproteins [62]. No such α-helix association was observed in r*Mro*UPO, but the described hydrophobic and polar interactions at the dimer interface could contribute to the dimerization mechanism in this enzyme, maintaining the dimeric structure of the C227A variant (as shown by SEC and analytical centrifugation). A comparison of the two dimeric arrangements found in r*Mro*UPO and r*Cvi*UPO is illustrated in Figure 7.

### 4.3. Some (Bio)Technological Implications

Owing to oxirane ring reactivity, fatty acid epoxides are of interest for the industrial production of biobased chemicals and intermediates [63,64] including binder ingredients as cross-linkable materials. UPOs catalyze the epoxidation of (poly)unsaturated fatty acids [65] without the drawbacks of chemical [66] and chemoenzymatic [67] epoxidation via peracids. Although r*Mro*UPO and r*Cvi*UPO engineering has been addressed to improve the epoxidation selectivity, monoepoxides are generally the main products obtained [34,35,36].

In this context, the strong improvement in diepoxide production (representing >50% of products after 98% conversion of linoleic acid by the K228stop variant of r*Cvi*UPO) is a result of industrial relevance, since diepoxides will confer the desired cross-linking properties to biobased epoxy binders. The C-terminal region removed by the K228stop mutation could not be solved in the r*Cvi*UPO crystal structure due to high mobility, as found for other UPOs [17]. However, its AlphaFold [51] modeling revealed that it extends directly over the heme access in the crystal structure (Figure S4). In this way, C-terminal tail removal in r*Cvi*UPO would facilitate the access of linoleic acid to the active site providing the structural basis for its improved diepoxidation activity.

Moreover, improved catalytic efficiency of the r*Cvi*UPO variants was observed for other substrates, including the bulky ABTS molecule. It had been suggested that dimerization would not affect the access to the heme cofactor in *Mro*UPO [57]. However, the C227A variant generated here, in spite of remaining a dimer as discussed above, completely lost its catalytic activity. This would be explained by a different disposition of the mutated C-terminal tail (lacking the stabilizing disulfide bond) at the entrance of the heme access channel.

### 4.4. Concluding Remarks

In conclusion, although additional interactions at the dimer interface seem to contribute to the observed UPO dimerization, as suggested by directed mutagenesis combined with SEC experiments, the solved crystal structures show the existence of the intermonomer disulfide bridge previously proposed in *Mro*UPO, and the parallel association between helix-12 of both r*Cvi*UPO monomers, as main elements of two different dimeric arrangements existing in short UPOs.

## Figures and Tables

**Table 1:** X-ray crystallographic data collection statistics.

	r*Mro*UPO	r*Cvi*UPO
Wavelength (Å)	0.9792	0.9792
Space group	C2221	P212121
Unit cell dimensions:		
a	100.3 Å	78.5 Å
b	107.4 Å	140.1 Å
c	186.2 Å	42.2 Å
Resolution (Å)	73.3–1.45	70.1–1.95
R_meas_^a^	0.094 (0.81)	0.191 (0.99)
R_pim_	0.037 (0.33)	0.057 (0.40)
I/σI	12.0 (2.3)	10.1 (2.4)
Completeness (%)	99.4 (99.5)	100 (100)
Redundancy	6.1 (5.8)	10.6 (6.2)
CC_1/2_^b^ (%)	99.7 (66.2)	99.4 (83.5)

Statistics for the highest-resolution shell are shown in parenthesis. **^a^** R_meas_ = Σ_hkl_.(n/n−1)^1/2^Σ_i_ |I_i_(hkl)-<|>(hkl)/Σ_hkl_Σ_i_I_i_(hkl), where I_i_(hkl) is the intensity measured for the ith reflection and <I>(hkl)) is the average intensity of all reflections with indices hkl. **^b^** CC_1/2_ is the correlation coefficient between two random half datasets.

**Table 2:** Refinement statistics.

	r*Mro*UPO	r*Cvi*UPO
Refinement:		
Resolution (Å)	73.3–1.45	70.1–1.95
No. unique reflections	167,101 (12,200)	33,334 (2439)
R_work_/R_free_	0.158/0.185	0.191/0.243
No. atoms:		
Protein	7276	3664
Ligand	172	86
Ion	4	2
Water	1066	268
B-factors (Å^2^):		
Protein	15.7	26.0
Ligand	12.6	20.3
Ion	8.38	14.97
Water	28.54	34.1
R.m.s deviations:		
Bond lengths (Å)	0.013	0.010
Bond angles (°)	1.85	1.53
Ramachandran:		
Favored (%)	96.41	94.21
Allowed (%)	3.17	5.57
Outliers (%)	0.42	0.22
PDB codes:	7ZBP	7ZCL

Values in parentheses refer to the highest resolution shells.

**Table 3:** Kinetic constants—*K*_m_ (μM), *k*_cat_ (s^−1^), and *k*_cat_/*K*_m_ (s^−1^·mM^−1^)—of wild *Mro*UPO [22], recombinant r*Mro*UPO and its C227A variant, and recombinant r*Cvi*UPO and its C235A and K228stop variants.

		*Mro*UPO	r*Mro*UPO	C227A	r*Cvi*UPO	C235A	K228stop
Veratryl alcohol	*K*_m_	279	54.2 ± 16.7	-	2940 ± 160	7240 ± 820	55,300 ± 10,000
	*k*_cat_	49	2.49 ± 0.16	0	2.24 ± 0.03	11.2 ± 0.4	56.4 ± 6.6
	*k*_cat_ */K*_m_	176	46 ± 12	-	0.75 ± 0.03	1.5 ± 0.2	1.0 ± 0.2
ABTS	*K*_m_	71	246 ± 3	-	239 ± 8 ^1^	87 ± 7	110 ± 15
	*k*_cat_	25	15.0 ± 3.3	0	157.2 ± 2.8	257.0 ± 4.9	209 ± 7
	*k*_cat_ */K*_m_	350	62 ± 14	-	656 ± 26	2970 ± 190	1900 ± 210
DMP	*K*_m_	133	206 ± 38	-	4930 ± 470	4500 ± 424	3720 ± 250
	*k*_cat_	70	66.2 ± 3.1	0	325 ± 11	812 ± 26	728 ± 16
	*k*_cat_ */K*_m_	530	320 ± 50	-	66 ± 5	180 ± 12	195 ± 10
H_2_O_2_	*K*_m_	3140	1880 ± 130	-	2250 ± 750 ^2^	420 ± 6 ^3^	680 ± 120 ^4^
	*k*_cat_ */K*_m_	24.2	25.8 ± 0.4	-	1120 ± 475	5170 ± 130	3930 ± 170

r*Mro*UPO constants were determined at optimal pH 4.6 for veratryl alcohol, pH 5.0 for ABTS, and pH 4.0 for DMP, with 5 mM H_2_O_2_; r*Cvi*UPO constants were determined at optimal pH 3 for veratryl alcohol oxidation by the native enzyme, pH 4.0 for ABTS and veratryl alcohol oxidation by variants, and pH 5.0 for DMP, with 1 mM H_2_O_2_. DMP was used as reducing substrate for H_2_O_2_ constants. ^1–4^ Substrate inhibition (with *k*_i_ values of 7860 ± 680, 1100 ± 360, 2550 ± 370, and 2140 ± 370 µM, respectively), and *K*_m_ and *k*_cat_/*K*_m_ calculated from Equation (3). Means and standard errors are provided for r*Mro*UPO, r*Cvi*UPO and variants.

**Table 4:** Fatty acid conversion, percentages of main products, and epoxidation yield in the reactions of linoleic acid with native r*Cvi*UPO and the short-tail K228stop variant (see Figure S5 for chromatographic profiles).

	Conversion			Products	(%)		Epoxidation
	(%)	12-Epoxy	9-Epoxy	Diepoxy	Hydroxy	OH-Epoxy	Yield (%)
r*Cvi*UPO	97	56	10	-	8	26	45
K228stop	98	14	16	51	13	6	68

The products from 30 min reactions of 0.4 µM enzyme in 50 mM phosphate (pH 7) with 20% acetone were extracted with methyl *tert*-butyl ether and analyzed by GC–MS as trimethylsilyl derivatives.

## Data Availability

All data underlying this article are available in the main publication and its Supplemental Materials online.

## Supplementary Materials

The following supporting information can be downloaded at: https://www.mdpi.com/article/10.3390/antiox11050891/s1, UV–visible spectra of r*Mro*UPO and r*Cvi*UPO resting states and complexes with CO (Figure S1), SDS-PAGE of r*Mro*UPO and r*Cvi*UPO under native and denaturing conditions (Figure S2), Sedimentation velocity profiles and *c(s)* distributions from analytical ultracentrifugation (Figure S3), Superimposition of the dimeric r*Cvi*UPO crystal structure and AlphaFold model showing the nonsolved C-tail (Figure S4), GC–MS analysis of linoleic acid reactions with native r*Cvi*UPO and its K228stop variant (Figure S5), different UPO heme pocket residues (Figure S6), and Heme channel opening in UPO crystal structures (Figure S7).
