# Supplementary material for: Structural Characterization of Two Short Unspecific Peroxygenases: Two Different Dimeric Arrangements
Source: Antioxidants (Basel). 2022 Apr 30;11(5):891. doi: 10.3390/antiox11050891 (PMC9137552; doi:10.3390/antiox11050891)
Supplement: Supplementary file 1 [file antioxidants-11-00891-s001.zip › antioxidants-1675293-supplementary.pdf]

antioxidants

MDPI

SUPPLEMENTARY MATERIALS

Structural characterization of two short unspecific peroxygenases: Two different dimeric arrangements

Dolores Linde [1‡], Elena Santillana [1‡], Elena Fernández-Fueyo [2], Alejandro González-Benjumea [3], Juan Carro [1], Ana Gutiérrez [3], Angel T. Martínez [1] and **Antonio Romero [1*]**

[1] Centro de Investigaciones Biológicas "Margarita Salas", CSIC, E-28040 Madrid, Spain. lolalinde@cib.csic.es (D.L.); esh@cib.csic.es (E.S.); jcarro@cib.csic.es (J.C.); atmartinez@cib.csic.es (A.T.M.); and romero@cib.csic.es (A.R.)

[2] Intravacc, Bilthoven, Utrecht, The Netherlands. elena.fueyo@intravacc.nl (E.F.-F.)

[3] Instituto de Recursos Naturales y Agrobiología de Sevilla, CSIC, E-41012 Seville, Spain. a.g.benjumea@irnas.csic.es (A.G.-B.); and anagu@irnase.csic.es (A.G.)

* Correspondence: atmartinez@cib.csic.es and romero@cib.csic.es, (+34 918373112)

‡ These two authors equally contributed to the work

This Supplementary Materials includes: UV-visible spectra of r*Mro*UPO and r*Cvi*UPO resting states and complexes with CO (**Figure S1**), SDS-PAGE of r*Mro*UPO and r*Cvi*UPO under native and denaturing conditions (**Figure S2**), Sedimentation velocity profiles and *c(s)* distributions from analytical ultracentrifugation (**Figure S3**), Superimposition of the dimeric r*Cvi*UPO crystal structure and AlphaFold model showing the non-solved C-tail (**Figure S4**), GC-MS analysis of linoleic acid reactions with native r*Cvi*UPO and its K228stop variant (**Figure S5**), Different UPO heme pocket residues (**Figure S6**) and Heme channel opening in UPO crystal structures (**Figure S7**).

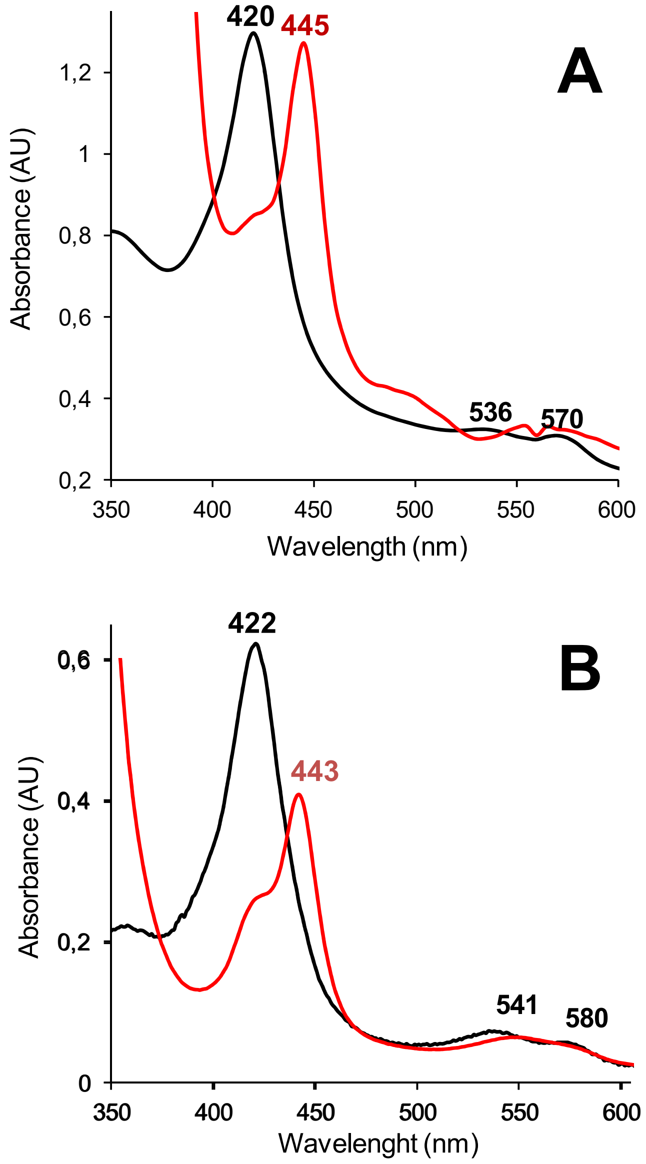

Figure S1. UV-visible spectra of r*Mro*UPO (**A**) and r*Cvi*UPO (**B**) resting states (*black*) and complexes (of reduced enzyme) with CO (*red*) where the main Soret band is displaced from 420-422 nm to 443-445 nm. Two minor bands are also observed in the 530-590 nm region (appearing at 536/570 and 541-580 nm in the spectra of the resting-state r*Mro*UPO and r*Cvi*UPO, respectively).

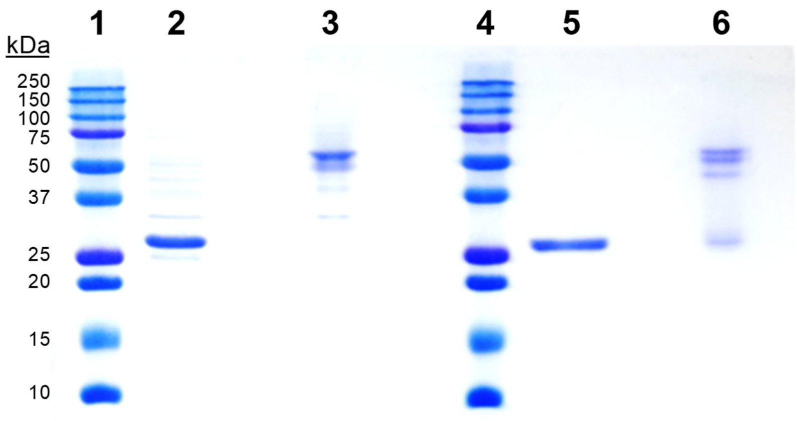

Figure S2. SDS-PAGE of r*Mro*UPO and r*Cvi*UPO under native and denaturing conditions (and standards). Monomeric forms of ~26-27 kDa are observed after heating at 100ºC with SDS and β-mercaptoethanol (lanes 2 and 5), while dimeric forms of ~50 kDa are observed without any treatment (lanes 3 and 6). Same protein amount (~1.7 µg) was loaded in each case. Molecular-mass standards from BioRad are included (lanes 1 and 4). SDS-PAGE was performed with 0.1% SDS containing 1% mercaptoethanol.

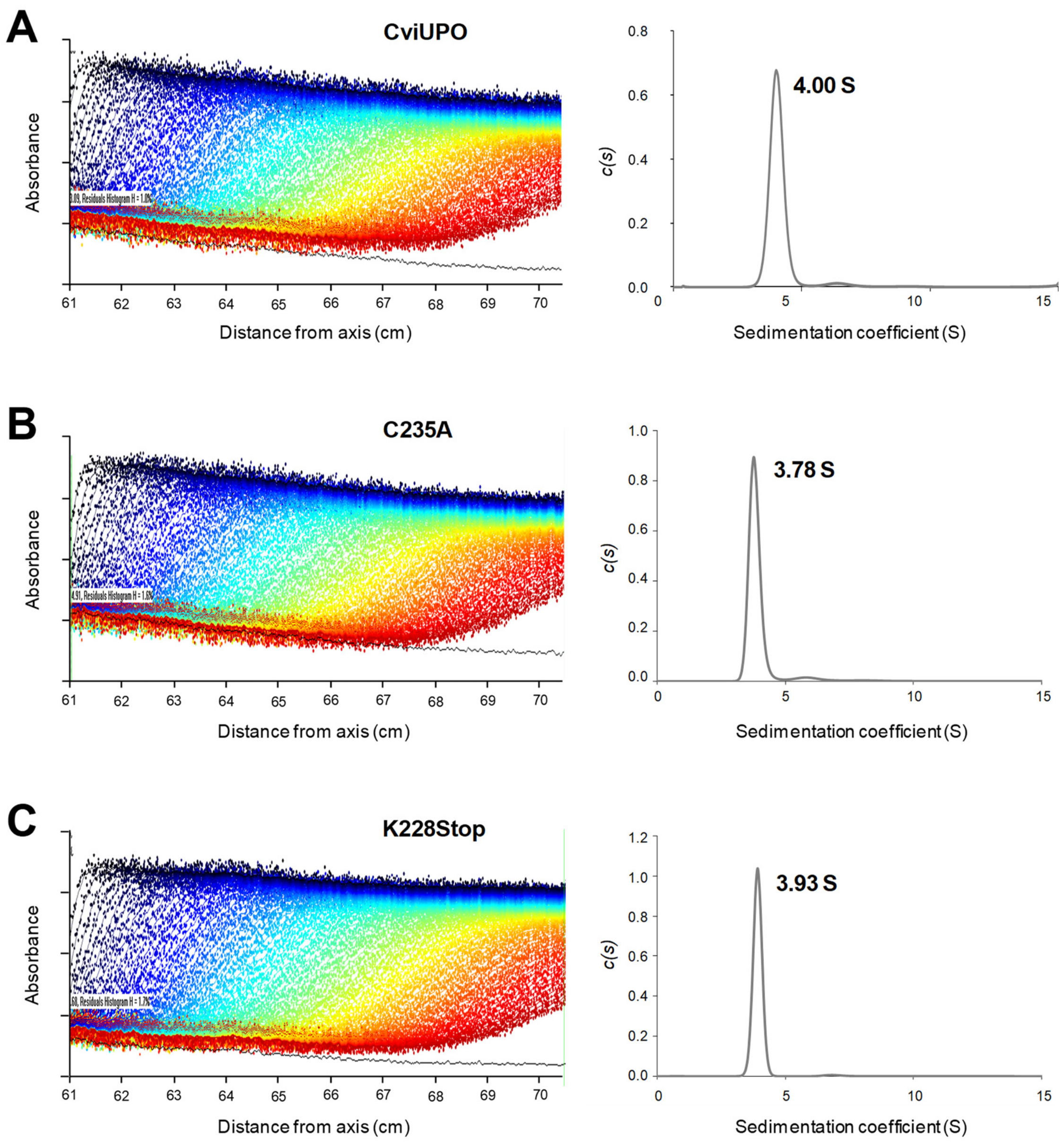

Figure S3. Sedimentation velocity profiles at 445 nm (*left*) and *c(s)* distributions (*right*) from analytical ultracentrifugation of native r*Cvi*UPO and its C235A and K228stop variants (**A-C**, respectively).

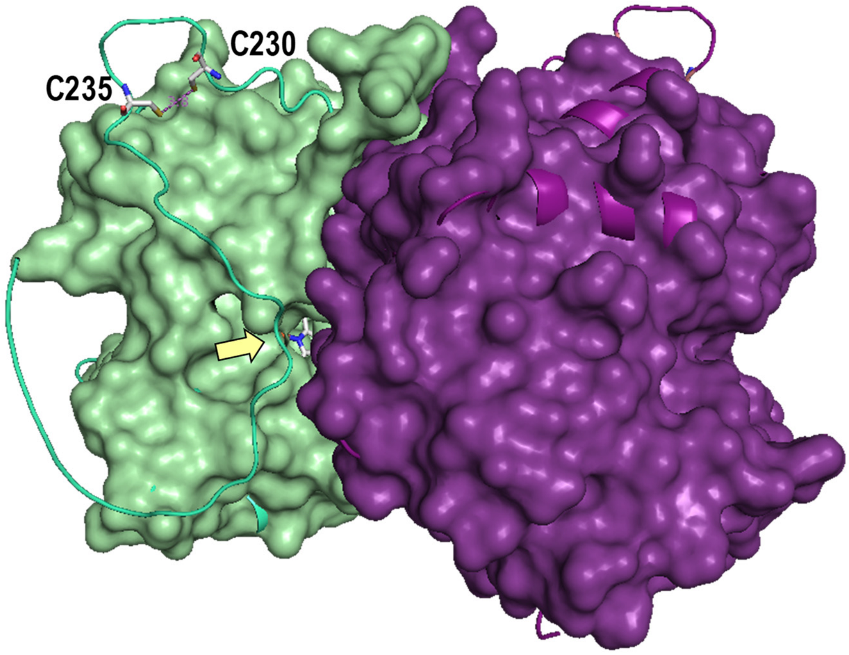

Figure S4. Superimposition of the dimeric r*Cvi*UPO crystal structure (PDB 7ZCL, solvent-access surface) and AlphaFold model showing non-solved C-tail with putative intramolecular C230-C235 bridge (visible in monomer A). As shown (yellow arrow) the modeled C-tail crosses in front of the channel leading to the heme cofactor (CPK-colored sticks) in the crystal molecular surface (visible in monomer A). For consistency with residue numbering in the wild enzymes (mature proteins isolated from fungal cultures), the introduced initial methionine was not considered for residue numbering in the recombinant enzymes.

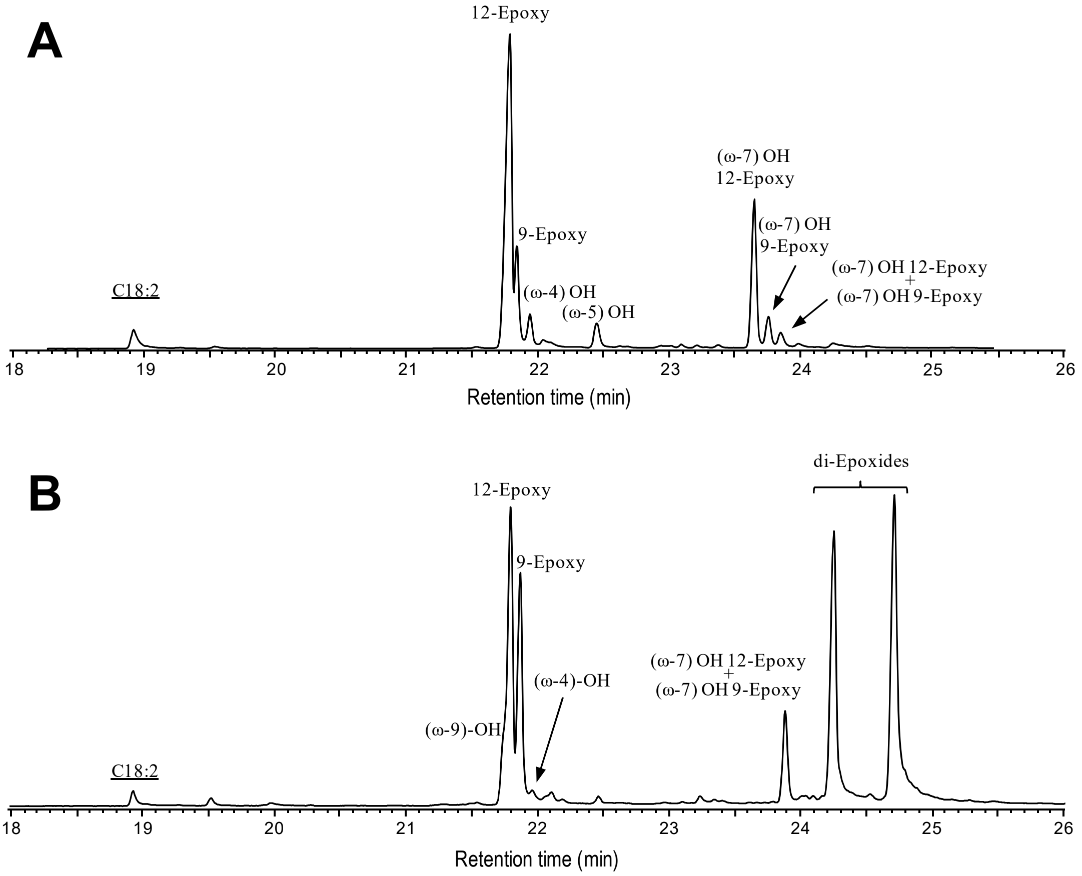

Figure S5. GC-MS analysis of linoleic acid (C18:2) reactions with native r*Cvi*UPO (**A**) and its K228stop variant (**B**). The reaction mixtures –which contained 0.1 mM substrate, 0.4 µM enzyme and 1.25 mM H_2O_2– were incubated for 30 min, extracted and derivatized before GC-MS analysis. The identified products include 9/12-monoepoxy-, minor (ω-4)/(ω-5)/(ω-9)-hydroxy-, (ω-7)-hydroxy-9/12-monoepoxy-, and diepoxy- derivatives (the two latter as several isomers, some of them in overlapping peaks).

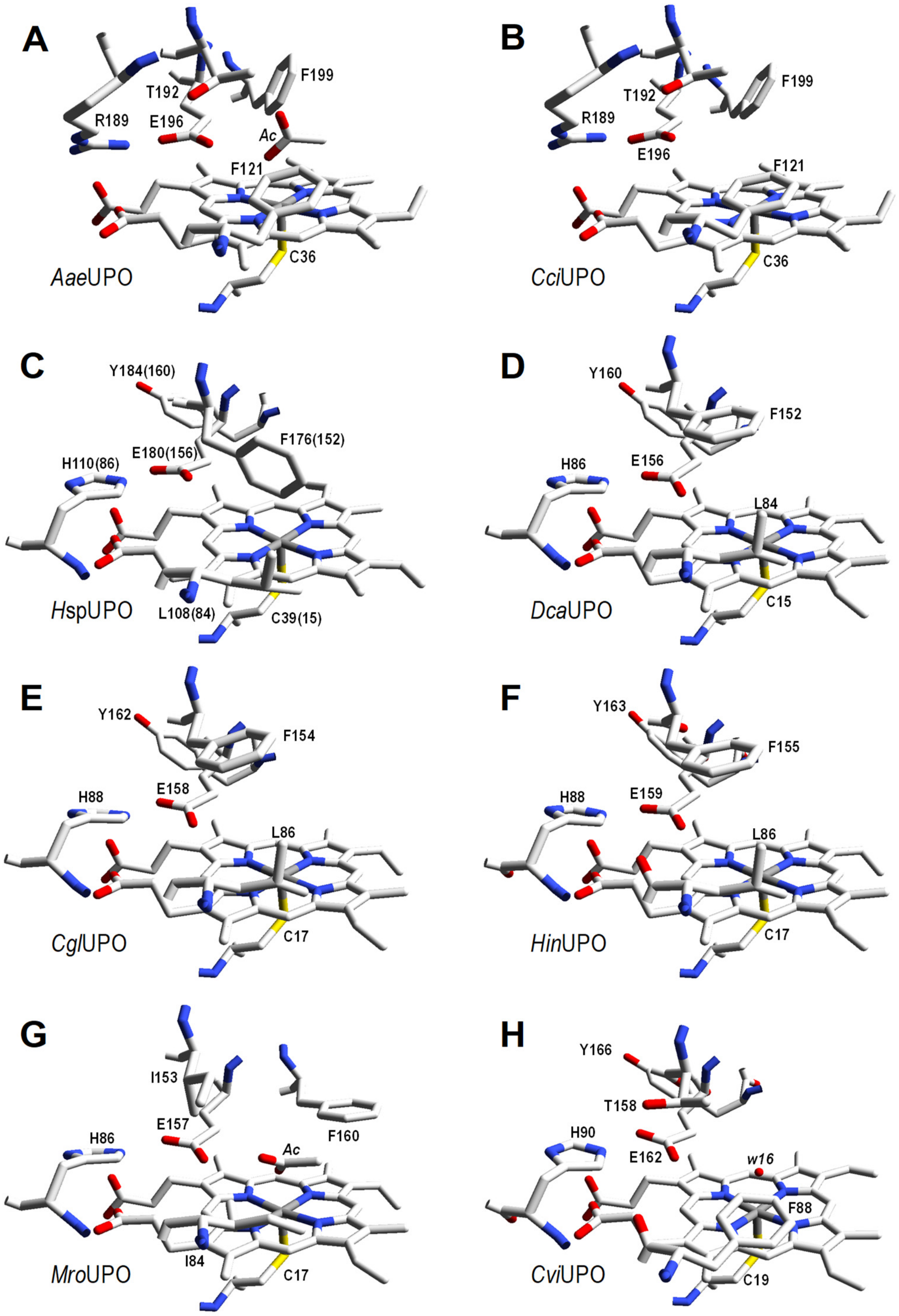

Figure S6. UPO heme pocket residues. **A)** *Aae*UPO. **B)** *Cci*UPO. **C)** *H*spUPO. **D)** *Dca*UPO. **E)** *Cgl*UPO. **F)** *Hin*UPO. **G)** *Mro*UPO. **H)** *Cvi*UPO. From crystal structures in **A** (2YP1)**, C** (7O1R)**, G** (7ZBP) and **H** (7ZCL) and homology models in **B**, and **D-F** (the r*Mro*UPO structure in **G** is superimposable with that of unpublished wild *Mro*UPO, 5FUJ/5FUK). Enzymes from basidiomycetes (**A,B,G** and **H**) and ascomycetes (**C-F**) are included that belong to the long (**A,B**) and short (**C-H**) UPO families. Numbering corresponds to the mature protein sequences except in **C**, where the signal sequence is included (Rotilio et al. 2021. ACS Catal. 11: 11511-25) (for comparison the mature r*Hsp*UPO residues are given in parentheses, according to the cleavage predicted in Mycocosm; https://mycocosm.jgi.doe.gov, accessed on 1 March 2022) .

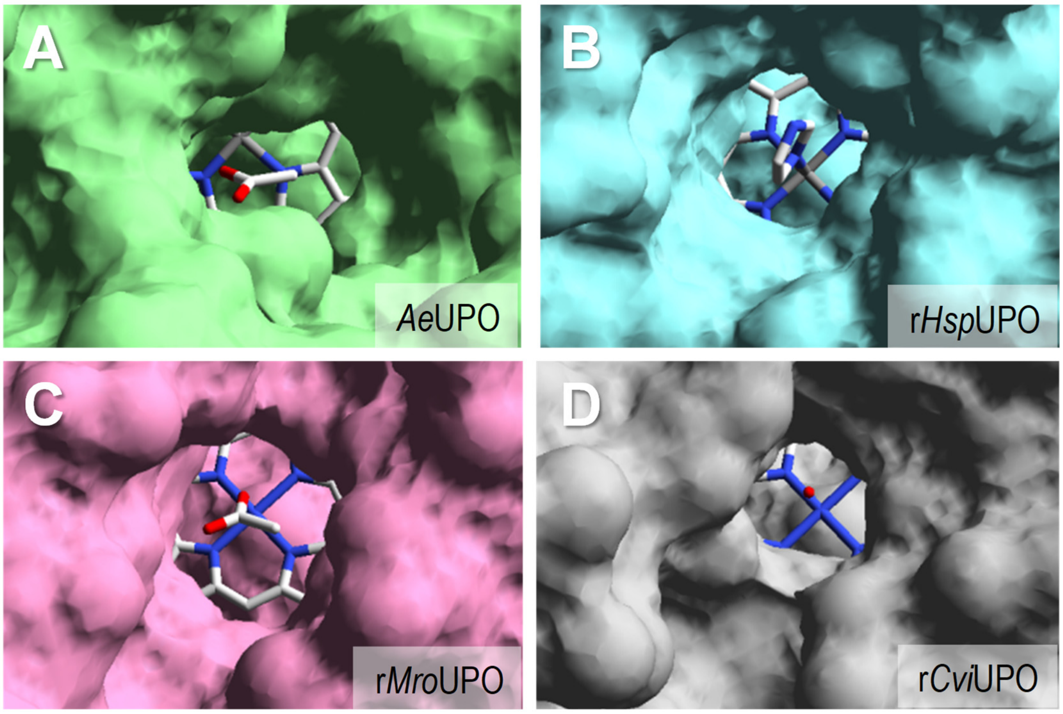

Figure S7. Heme channel opening in UPO crystal structures. **A)** *Aae*UPO (2YP1). **B)** r*Hsp*UPO (7O1R). **C)** r*Mro*UPO (7ZBP). **D)** r*Cvi*UPO (7ZCL). Acetate (**A** and **C**) or water (**D**) oxygen, and imidazole nitrogen (**D**) atoms are close to the sixth coordination position of the heme iron (3.0 Å distance in *Aae*UPO and 2.2-2.5 Å distances in the rest).
